# Supplementary material for: Depressive Symptoms and Gut Microbiota after Bowel Preparation and Colonoscopy: A Pre–Post Intervention Study
Source: Microorganisms. 2024 Sep 27;12(10):1960. doi: 10.3390/microorganisms12101960 (PMC11509487; doi:10.3390/microorganisms12101960)
Supplement: Supplementary file 1 [file microorganisms-12-01960-s001.zip › microorganisms-3189428-supplementary.pdf]

Supplementary Table S1. Bowel preparation adequacy and colonoscopy indications and findings.

	Complete cases (N=59)	Lost to follow up (N=20)	All cases (N=79)
**Bowel preparation adequacy, yes n(%)** [a]			
Fair	15 (25%)	5 (25%)	20 (25%)
Good	37 (63%)	11 (55%)	48 (61%)
Excellent	7 (12%)	4 (20%)	11 (14%)
**Indication for colonoscopy, yes n(%)**			
Positive faecal occult blood test	24 (41%)	6 (30%)	30 (38%)
Surveillance colonoscopy [c]	19 (32%)	1 (5%)	20 (25%)
Other symptoms [e]	18 (31%)	8 (40%)	26 (33%)
Change in bowel habits [b]	5 (9%)	7 (35%)	12 (15%)
Iron-deficiency anaemia	5 (9%)	1 (5%)	6 (8%)
Suspected lesions [d]	2 (3%)	0 (0%)	2 (3%)
**Colonoscopy outcome, yes n(%)** [f]			
No abnormalities detected	12 (20%)	7 (35%)	19 (24%)
Diverticular disease	18 (31%)	4 (20%)	22 (28%)
Polyps	37 (63%)	9 (45%)	46 (58%)
Other [g]	16 (27%)	8 (40%)	24 (30%)

Notes:

[a] *Based on an overall Boston Bowel Preparation Scale score.*

[b] *Change in bowel habit(s) includes frequent stools, diarrhoea, faecal incontinence.*

[c] *Surveillance includes for family history, previous polyps, previous gastric surgery.*

[d] *Clinically or radiologically detected lesions included thickened area on scans and possible rectal mass detected; detected lesions were not associated with a cancer diagnosis.*

[e] *Other symptoms included abdominal pain, peri-rectal bleeding, haemorrhoids, anorectal pain, weight loss, vomiting, bloating, pain on defecation.*

[f] *Some participants received more than one diagnosis after colonoscopy, therefore percentages exceed 100%.*

[g] *Other outcomes included haemorrhoids, granular mucosa, and muscle hypertrophy.*

Supplementary Table S2. Unadjusted and adjusted models of the change in beta-diversity of faecal samples collected one week before and one month after bowel preparation and colonoscopy using the Aitchison distance metric.

	**Aitchison distance**				
Predictors	*Df*	*Sum of squares*	*R^2*	*F*	*p-value*
**Unadjusted model**					
Time point	1	1975	0.004	0.441	**0.001**
Residual	116	519339	0.996		
Total	117	521314	1.000		
**Adjusted model**					
Age (years)	1	7610	0.015	1.749	0.246
Sex (male)	1	7228	0.014	1.661	0.604
BMI	1	7549	0.015	1.735	0.374
Diet quality	1	8891	0.017	2.043	0.028
IBS (yes)	1	5302	0.010	1.219	0.287
Time point	1	1976	0.004	0.454	0.001
Residual	111	482935	0.926		
Total	117	521314	1.000		

Note: Age at time of recruitment; BMI calculated as weight (kilograms)/height(metres)^2 at time of recruitment; Diet quality measured using a Simple Dietary Questionnaire based on previous studies(37).

Abbreviations: BMI, body mass index; CI, confidence interval; Df, degrees of freedom; IBS, irritable bowel syndrome.
